# Bacteriophages as Agents for Plant Disease Control: Where Are We After a Century?

**DOI:** 10.3390/v17081033

**Published:** 2025-07-23

**Authors:** Manoj Choudhary, Ibukunoluwa A. Bankole, Sophia T. McDuffee, Apekshya Parajuli, Mousami Poudel, Botond Balogh, Mathews L. Paret, Jeffrey B. Jones

**Affiliations:** 1 Indian Council of Agriculture Research, New Delhi 110001, India; manoj04444@gmail.com; 2 Department of Plant Pathology, University of Florida, Gainesville, FL 32611, USA; i.bankole@ufl.edu (I.A.B.); smcduffee@ufl.edu (S.T.M.); aparajuli@ufl.edu (A.P.); poudel.mousami@ufl.edu (M.P.); botond.balogh@gmail.com (B.B.); paret@ufl.edu (M.L.P.)

**Keywords:** bacteriophage, antibiotic resistance, biocontrol, challenges

## Abstract

The rise in antibiotic-resistant bacteria has made the management of bacterial diseases increasingly challenging. As a result, bacteriophages have gained attention as a promising alternative to antibiotics for combating bacterial pathogens. However, the usage of phages as biocontrol agents faces many challenges, including environmental stability, delivery efficiency, host specificity, and potential bacterial resistance. Advancements in genetic engineering and nanotechnology have been explored to enhance the stability, efficacy, and adaptability of phage-based treatments. In this review, we discuss the key barriers to the effective implementation of phage therapy and highlight innovative strategies to overcome these challenges. By addressing these limitations, this review aims to provide insights into optimizing phage-based approaches for widespread therapeutic and biocontrol applications.

## 1. Introduction

**Figure 1:**
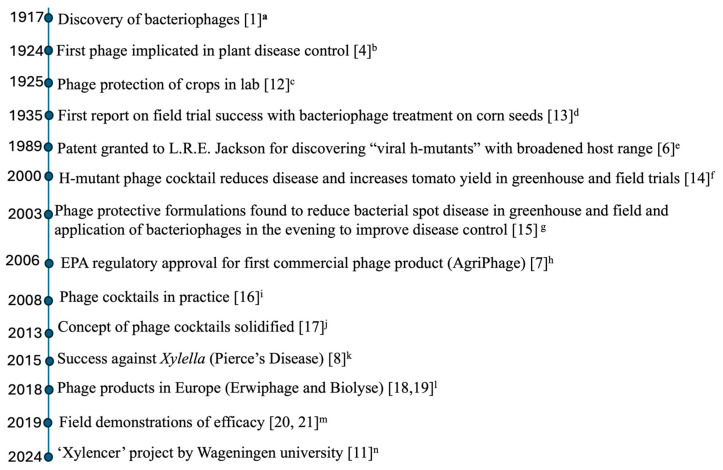
Chronological events in the use of bacteriophages for control of plant diseases (1917–2024). ^a^ Twort first independently characterized bacteriophage in 1915. In 1917, Felix d’Herelle independently discovered bacteriophage and described the lytic principle. ^b^ Mallmann and Hemstreet showed that a filtrate from rotting cabbage (containing phages) could inhibit the black rot pathogen *Xanthomonas campestris pv. campestris*. ^c^ Kotila and Coons [12] demonstrated that phages could prevent soft rot in potato and carrot slices caused by *Pectobacterium* species (then *Bacillus carotovorus*), indicating phage potential to curb post-harvest rot. ^d^ Thomas [13] conducted the first phage field trials by treating corn seeds with phage to control Stewart’s wilt (caused by *Pantoea stewartii*). The phage treatment cut disease incidence from 18% in controls to ~1.5%, a remarkable early success. ^e^ L.R.E. Jackson discovered “viral h-mutants”–phage mutants with broadened host range, that could kill plant-pathogenic *Pseudomonas syringae* (cause of bean halo blight, etc.), including strains resistant to parent phages. This work led to a patent in 1989 describing mixtures of such host-range mutant phages to prevent bacterial plant diseases and frost injury. ^f^ Flaherty et al. [14] published a study evaluating host-range mutant (h-mutant) bacteriophage cocktails to control bacterial spot (*Xanthomonas campestris* pv. *vesicatoria*) on tomato. In both greenhouse and field conditions (1997–1998), phage treatments significantly reduced disease incidence and improved plant vigor. The phage-treated plants showed 17–25% reduction in disease severity and up to 24% increase in extra-large fruit yield compared to untreated controls, outperforming traditional copper-based bactericides. This work was one of the earliest and most compelling demonstrations of phage cocktail efficacy in real-world crop production. ^g^ Balogh et al. [15] used phage protective formulations found to reduce bacterial spot disease in greenhouse and field, and application of bacteriophages in the evening to improve disease control. ^h^ AgriPhage received full U.S. Environmental Protection Agency approval, making it the first bacteriophage-based pesticide for plant disease. Developed by Omnilytics Inc., AgriPhage initially targeted bacterial spot and speck in tomatoes and peppers (*Xanthomonas campestris* pv. *vesicatoria* and *Pseudomonas syringae* pv. *tomato*). This milestone marked the transition of phage therapy from lab/field trials to a regulated commercial agricultural product. ^i^ Researchers began demonstrating that mixtures of phages (cocktails) improve efficacy and overcome the narrow host range of single phages. For example, in Florida, Balogh et al. [16] applied a four-phage cocktail (formulated in a protective skim-milk carrier) to citrus seedlings and achieved ~50–60% reduction in disease severity for citrus canker and bacterial spot. This was among the first modern studies showing that phage cocktails can effectively suppress plant diseases in greenhouse conditions. ^j^ Chan et al. [17] and others formally proposed phage cocktails as a necessity for robust biocontrol, especially in heterogeneous field environments where a single phage often can’t infect all pathogenic strains. ^k^ A breakthrough in managing Pierce’s disease of grapevines (caused by *Xylella fastidi*osa) was reported by Das et al. [8]. A cocktail of four lytic phages was injected into diseased grapevines, significantly reducing *Xylella* levels and halting disease progression. This study demonstrated phage cocktails as a viable strategy against a lethal, systemic plant disease, garnering wide attention. ^l^ The first bacteriophage products for agriculture emerged in Europe. In Hungary, an “Erwiphage™ Plus” cocktail targeting fire blight (*Erwinia amylovora*) was released in 2018 [18]. Around the same time, a UK company (APS Biocontrol) introduced Biolyse^®^, a phage-based solution to prevent soft rot in stored potatoes [19]. These mark the entry of phage biocontrol into the European market, despite regulatory hurdles. ^m^ Multiple field trials in the late 2010s validated that phage cocktails can control plant pathogens under real-world conditions. For example, in 2019, Wang et al. [20] showed an 80% reduction of tomato wilt caused by *Ralstonia solanacearum* in field plots using a four-phage cocktail. Similarly, Carstens et al. [21] showed that a six-phage mix against potato soft rot/blackleg (*Pectobacterium atrosepticum*) cut disease incidence by ~60%. These studies confirmed that carefully formulated cocktails can significantly reduce plant disease in both greenhouse and field settings. ^n^ In 2024 the “Xylencer” project (Wageningen University) engineered phages to combat *Xylella* in olive trees, showing promising results against this quarantine pathogen.

Bacteriophages were first independently characterized by Felix d’Herrelle and Frederick Twort (Figure 1) over a century ago [1,2]. The name, coined by d’Herelle, originates from bacteriophage, meaning ‘bacteria-eater,’ derived from the Greek phago, which means ‘to eat’ or ‘to devour’ [1]. Since then, their potential applications have remained a subject of ongoing scientific investigation and discourse (Figure 1). Due to their potent antibacterial properties, phages were rapidly adopted to prevent and treat both plant and human infections (a clinical approach commonly called ‘bacteriophage therapy’ or ‘phage therapy’) shortly after their discovery. Phage therapy emerged as a promising approach for combating bacterial diseases in the early 20th century, particularly in hot and humid climates, where such diseases often caused devastating crop losses [3,4,5].

The first bacteriophage patent for control of bacterial plant diseases was granted to L. R. E. Jackson [6] and identified broad-range viral h-mutants as effective phages against plant pathogenic bacteria, aiding in the prevention and control of plant diseases and ice nucleation (Figure 1). Several phage-based products have since received approval from the U.S. Environmental Protection Agency (EPA). The first registered product for the use of bacteriophages for control of bacterial plant diseases, Agriphage^TM^, targets *Xanthomonas campestris* pv. *vesicatoria* and *Pseudomonas syringae* pv. *tomato* on peppers and tomatoes [7]. Another notable example is a phage cocktail developed to combat Pierce’s disease in grapevines caused by *Xylella fastidiosa* subsp. *fastidiosa* [8]. However, despite these advancements, field studies often report inconsistent results due to environmental challenges such as weather variability, as well as the need to optimize delivery methods and timing for effective biocontrol. To address these challenges, standardized protocols and strategies have been recommended to improve the reliability and efficacy of phage-based applications [9,10]. An interesting project, Xylencer, from Wageningen University leverages synthetic biology and bacteriophages to target *Xylella fastidiosa* and has shown promising results [11]. Additionally, growing regulatory restrictions on agricultural antibiotic use have heightened interest in utilizing bacteriophages to manage antimicrobial resistance in agriculture.

### 1.1. What Are Bacteriophages?

Bacteriophages, commonly referred to as phages, are viruses that specifically target bacterial hosts and exhibit typical viral characteristics. They cannot replicate independently and must rely on a bacterial host for reproduction. Phages are believed to be among the oldest known biological entities, dating back approximately 3 billion years [22]. They are also the most abundant organisms in the world, with ocean water containing approximately 10^7^ phage particles per milliliter and the biosphere harboring an estimated ∼10^31^ virus-like particles, outnumbering bacteria by a factor of ten [23,24].

Phages typically measure between 50 and 200 nm and carry genetic instructions that facilitate rapid and efficient replication. The ability of a bacteriophage strain to infect different bacterial strains defines its host range and varies widely [25]. Some phages exhibit a narrow host range, infecting a few bacterial strains, while others have a broad host range, capable of infecting multiple strains within a bacterial species or different bacterial species within the same genus or even across different genera [25,26,27,28]. Cross-genera infection is rare and is usually limited to closely related bacteria. Host range plays a pivotal role in determining the efficacy of phage-based applications [25].

Despite their microscopic size, phages are visible under electron microscopy, showcasing a diversity of shapes and structures. The majority of environmental phages belong to the order *Caudovirales*, which are characterized by double-stranded DNA (dsDNA) and tails. Morphologically, these phages are classified into three main types based on their tail structures: *Siphoviridae* (long, non-contractile tails), *Myoviridae* (contractile tails consisting of a sheath and a central tube), and *Podoviridae* (short, non-contractile tails) (Table 1) [29,30].

### 1.2. Mechanism of Lytic Phages

**Figure 2:**
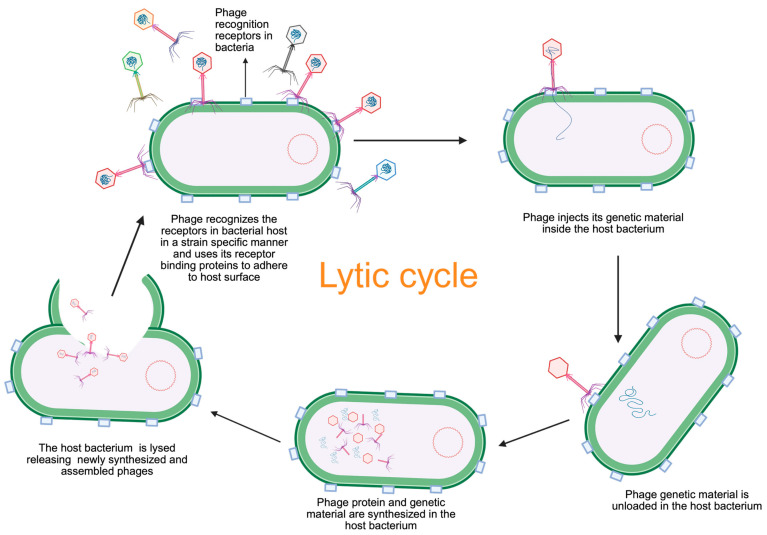
Lytic lifecycle of bacteriophage. It begins with specific recognition of a susceptible bacterial host, followed by adsorption to the bacterial surface. Phages are strain-specific—although multiple phages may be present in the environment, only those capable of recognizing a given bacterial strain can bind to it. (Note that the red-colored phage particle attaches to the receptor on the bacterial cell, while the blue-colored phage particle does not attach). Upon successful adsorption, the phage injects its nucleic acid into the host cell, initiating replication of the phage genome and synthesis of viral components. These components are assembled into mature virions, which are then released through lysis of the bacterial cell. The released phages can subsequently infect new bacterial hosts.

Most bacteriophages used in phage therapy predominantly follow a lytic lifecycle (Figure 2), characterized by the rapid infection and destruction of host bacterial cells [66]. The process begins with the phage attaching to specific receptors on the host cell surface in two distinct stages: an initial reversible interaction followed by irreversible binding. Once firmly attached, the phage employs lytic enzymes to degrade the host cell wall, facilitating the injection of its genetic material into the host cytoplasm [67]. Inside the host, the phage genome takes control of cellular processes, redirecting them from their normal functions to support viral replication and protein synthesis. This includes degradation of the host genome, replication of phage DNA, and production of viral proteins, including capsids. These components are then assembled into mature phage particles. Finally, lysis of the host cell occurs, driven by key phage proteins. Holins disrupt the host cell membrane, creating pores, while endolysins degrade the peptidoglycan layer of the cell wall [68]. This compromise of membrane integrity and loss of selective permeability causes osmotic imbalance, leading to cell lysis. The newly formed phage particles are subsequently released to infect other host cells, perpetuating the lytic cycle [69].

While lytic phages are typically favored in phage therapy for their immediate bactericidal effect, some lytic phages may be temperate and capable of entering a lysogenic lifecycle under certain conditions. This is a concern, as temperate phages integrate into the host genome and replicate alongside the bacterium, often without destroying it. Temperate phages pose several risks: they can carry toxin genes or pathogenicity islands that enhance bacterial virulence, participate in generalized or specialized transduction that facilitates horizontal gene transfer, and confer superinfection immunity to the lysogenized host, making it resistant to further phage infection [70]. These features can undermine the therapeutic efficacy of phage therapy and potentially worsen clinical outcomes. Thus, obligately lytic phage, those that do not undergo lysogeny, are preferred for clinical applications. The presence of lysogeny in therapeutic phages must therefore be carefully screened and avoided to mitigate these risks [71].

## 2. A Renewed Interest in Phage Therapy

The resurgence of interest in phage therapy is largely driven by concerns over rising antibiotic resistance in an era where traditional antibiotics are becoming less effective [72,73]. While a few phage-based treatments have been developed, their widespread clinical use remains limited. The growing resistance to antibiotics primarily stems from their excessive and improper use, creating selective pressures that drive bacterial adaptation [74].

Despite their potential for treating bacterial infections, phage therapy has yet to see widespread adoption. What are the key obstacles preventing its success, and can they be overcome? In this review, we explore the challenges hindering effective therapeutic use of phages and evaluate their prospects for future development.

### 2.1. Phage Survival and Persistence

Phage viability can be affected by exposure to a variety of environmental parameters, with the most detrimental parameter being ultraviolet (UV) radiation. UV radiation, particularly UV-B (280–315 nm), has been shown to cause significant damage to the DNA of bacteriophages, thereby impairing their ability to replicate [75,76]. Ozone depletion has led to an increase in UV-B intensity, with each 1% reduction in ozone concentration resulting in a 1.2% increase in UV-B levels. According to a 1994 report from the Palmer Station in Antarctica, seasonal stratospheric ozone depletion increased the UV-B radiation reaching terrestrial Antarctic habitats by up to 50%. This was also accompanied by shorter bursts of UV-B penetrating throughout the rest of the atmosphere [77]. An infective phage must contend with this increased UV-B exposure when it is applied to a potential host. UV-C radiation can have similar effects as UV-B, but it is thought to also affect phage adsorption to the host cells [78]. Overall, though, UV exposure of any kind generally reduces bacteriophage longevity. For instance, *Erwinia amylovora* phage Y2 exhibited a 99.79% reduction in PFUs after 5 min of UV exposure [79], while *Dickeya solani* phages became undetectable after 10 min. Similarly, the population of *Xanthomonas axonopodi*s pv. *citrumelo* phage declined from 10^6^ to 0 PFUs within 4 h when sprayed on tomato leaves [80].

To mitigate the adverse effects of UV radiation, phages can be applied during evening hours [10]. Studies have demonstrated that phage application after sunset significantly extends their persistence within the phyllosphere, providing an extended window for bacterial infection and control [81]. This strategy underscores the importance of optimizing phage application timing to maximize their efficacy in agricultural systems. Mixing bacteriophage suspensions with various compounds (i.e., formulations) has been used to protect phage longevity upon UV exposure. Balogh et al. [15] demonstrated increased phage longevity and efficacy when using formulations containing (i) pregelatinized corn flour (PCF) and sucrose; (ii) casecrete, sucrose, and PCF; and (iii) skim milk and sucrose (M + S). Another study investigated using readily available and natural sources as protective formulations for infective phages [82]. These formulations included extracts from carrot, red pepper, beetroot, casein, and soy peptone, as well as astaxanthin, aromatic amino acids, and Tween 80. All these compounds were found to significantly increase infective phage particle half-lives when irradiated with UV. In general, compounds with the ability to absorb detrimental UV can make good phage protectants.

In addition to UV radiation, phages are subject to hostile environmental conditions in the phyllosphere (leaf surface of a plant) and rhizosphere (region surrounding the roots). In the phyllosphere, phages are subjected to adverse conditions such as fluctuating temperature that may increase desiccation, chemicals such as copper pesticide residues that may be present on plant surfaces [80,83], and rain-induced leaching that reduces the number of phages on the leaf surface [84]. These issues prevent phages from coming into contact with potential hosts. Strategies to limit UV damage to phages are also generally effective at reducing damage from these environmental challenges, such as using protective formulations [81,85] or applying phages in the evening or at dawn. Another strategy is to maintain phage populations using propagating bacterial strains [10]. Bacteriophages mixed with a non-pathogenic bacterial species were used to enhance phage persistence on the leaf surface and were shown to improve biocontrol of black rot disease of broccoli plants [58].

In the rhizosphere, there is high heterogeneity in the soil due to variation in soil particle size and composition, soil biota, and microbiome diversity [86], which influences the movement or diffusion of phages through the soil. For instance, sandy soils with larger particles have more chances of phage movement owing to greater pore space and better water flow than poorly structured soils, like clay soils. Poor distribution and dispersal of phages were shown to create a barrier that prevented phages from contacting their host bacteria and, hence, were ineffective for disease control [87]. The stability of phages in the soil is also driven by temperature [88], soil moisture [89], and plant root exudates or litter composition [83]. Stability of phages can also be affected by anthropogenic factors such as continuous cropping, which could affect the abundance of the viral community in the soil [90]. Continuous cropping practices allowed for viral priming and affected soil viral abundance, where viruses remaining in the soil previous season adapted to infect the juvenile rhizosphere of the hosts in the next cropping season [89]. In contrast, crop rotation reduced viral priming activity in the rhizosphere [83]. In the context of soil phage therapy, this could influence a co-evolution and persistence of phage in the absence of their host to closely related host strains found in the rhizosphere [85]. Applying phages as seed coating can also improve phage persistence in the rhizosphere, especially for seed-borne diseases. Coating *Xcc*-contaminated brassica seeds with phages reduced the bacterial titer between 1 and 2 log units [59]. Likewise, maize seeds infected with *Clavibacter michiganensis subsp. nebraskensis* (*Cmn*) that causes Goss’ wilt were coated with phages and polymer stabilizers such as polyvinyl alcohol (PVOH), and the phage treatment was shown to reduce the bacterial titres of the pathogen by 76% on the seed surface and 51% in internally infected seed and 78% in the seedling tissue [91]. Coating of melon seeds with phage ACPWH resulted in 95% germination and 95% survival of the seedlings after soil inoculation with the bacterial fruit blotch pathogen, *Acidovorax citrulli,* as compared to 13% germination and 0% survival in non-coated seeds (Table 1) [31]. These studies show bacteriophages can persist on seeds even after germination without any negative effects on plants [58]. However, this could be limited to seedborne diseases and be effective against early stages of bacterial infection. The combination of seed coating and soil drenching of the phage could provide a long-lasting protection against the bacterial pathogen.

### 2.2. Phage Storage Challenges Under Laboratory and Commercial Conditions

In addition to UV radiation, many other environmental parameters can affect phage survival during storage and affect biocontrol efficacy. Temperature is one of the most important factors of successful phage storage, as elevated temperatures can adversely affect phage viability and infectivity [92]. Phages are therefore typically stored at low temperatures at 4 °C, −20 °C, or −80 °C [93], with increased longevity at the lowest temperatures. Other important considerations are the techniques used to make a phage suspension, storage media composition, conditions of the storage area, and methods used to apply the phages to the host cells [10,92]. In general, keeping phages cold and away from light is sufficient to maintain phage survival; however, the composition of the storage medium can also affect phage viability. Under experimental conditions, phages are typically stored in SM buffer. This buffer includes Tris-HCI, a compound that is not approved by the FDA for use as a food additive [94]. While this allows phages to be stored stably for long periods for experimental purposes, it cannot be used commercially for food applications, limiting its use for controlling plant pathogens. Many other potential storage media cannot be added to food due to their effect on the food’s aesthetic or practical characteristics, including flavor and odor [95]. Other potential food-safe media options do not seem to significantly affect phage survival and viability, and thus still require cold temperatures for continued viability [95]. These limitations can make it difficult to store phages. One option to get around some common storage issues is to store phages as a dry powder through lyophilization, or by freeze drying, which eliminates the need for temperature control during storage and shipping [96]. However, this method is costly and requires special equipment [95]. Another method to increase phage longevity during storage is to force the phages to undergo adaptive evolution in response to thermal stress, resulting in phage strains adapted to thermal stress and therefore making them easier to store [92].

### 2.3. Phage Host Range and Selection of Phage Candidates for Therapy

The high degree of specificity of phages in phage therapy is one of the advantages of managing plant bacterial diseases, by sparing beneficial microbes and maintaining the microbial ecosystem (Table 1). Some phages are so specific that they can only infect one bacterial species or even a few strains within a species [17,27]. However, this limits the use of these phages in targeting different phytopathogens in the field. A phage host range profile can be subjectively determined by the spectrum of strains that can be successfully infected and killed by the phage. The lysis of these strains is determined by the specificity of a phage’s host binding proteins that allow for attachment of the phage to the bacterial cell, and molecular interactions between the phage and bacterial cell, which lead to the injection of the phage genetic material and hijacking of the bacterial metabolism and production of new offspring [25,97]. Developing a phage biocide that eliminates every strain of a particular bacterial species can be an adsorption challenge. In recent times, to mitigate this challenge, multiple phages are often mixed, creating phage cocktails to target different pathogens of either different species causing similar disease or different strains in one species [14]. Several studies have shown the effectiveness of phage cocktails against plant bacterial pathogens. For example, phage combinations consisting of four phage types isolated from tomato fields decreased the incidence of bacterial wilt disease caused by *Ralstonia solanacearum* by up to 80% in field experiments [20]. In a study by Carstens et al. [21] a six-phage cocktail reduced both disease incidence and disease severity of black leg disease of potato stems by 61% and 64%, which is caused by *Pectobacterium atrosepticum* (Table 1). In addition, phage cocktail formulations containing several phages have protected plants against plant pathogenic bacterial species such as *Xylella fastidiosa* [8], *Xanthomonas campestris* pv. *vesicatoria* [60,98], *Xanthomonas axonopodis* pv. c*itrumelo* [59]*, Xanthomonas axonopodis* pv. c*itri* [59], *Xanthomonas campestris* pv. *pelargonii* [57]*, Pseudomonas syringae* pv. *porri* [42]*, Dickeya solani* [99], and *Xanthomonas axonopodis* pv. *allii* [56]. In addition, the commercially available phage mixture product, AgriPhage^TM^, is effective against several bacterial diseases caused by *Xanthomonas campestris* pv. v*esicatoria, Pseudomonas syringae* pv. t*omato, Erwinia amylovora, Clavibacter michiganensis* subsp. m*ichiganensis, Xanthomonas arboricola* pv. *pruni*, and *Xanthomonas arboricola* pv. *juglandis* [100].

For an effective phage cocktail application, a cocktail of lytic phages isolated from various sources, with diverse receptor binding proteins and strong adsorption, should be used to avoid or minimize resistance development [41,47]. Prior to phage cocktail preparation, each required phage host range should be determined, including its genomic features, application requirements, and efficiency against pathogens [17]. This is to avoid inefficiency and to minimize targeting potentially beneficial bacteria. Phages in a cocktail may also have a synergistic effect, where one phage may increase and augment the virulence of the other phages against the growth of the target bacteria [56,101]. Hence, careful attention should be given to each of the phages used in cocktails. In addition, due to the complexity of plant pathogen interactions, it should be emphasized that a single multidimensional cocktail for all bacterial phytopathogens may not be plausible to develop. Constant surveillance of emerging phage-resistant bacteria and modification of phage cocktail formulations should be performed as needed to ensure the effective killing of target pathogens [102]. Even in AgriPhage^TM^, they constantly monitor the phage population and update their product [100].

### 2.4. The Difficulty of Applying Phages Uniformly over a Large Area

Application over large areas requires a higher volume of phage. Phage titers of 1 × 10^6^ to 1 × 10^10^ PFU/mL have been used for plant disease control (Table 1). Maintaining a high phage titer is essential for effective disease control. While achieving this in small-scale applications may be relatively straightforward, large-scale deployment in fruit orchards, ranging from 29 to 49,535 acres, would require substantial phage volumes [103,104]. For example, one quart of AgriPhage—Tomato Spot/Speck- is recommended for one acre and costs approximately 50 USD [105]. Keeping in mind, phages should be applied repeatedly due to their instability in harsh environmental conditions. The application to large farms over a long time may not be viable. Although there has been modeling and research on lowering the cost of production of phages, commercial applications are still expensive when dealing with large acreages of farms.

According to Vu and Oh [106], bacteriophages are mostly applied in the rhizosphere through soil drenching, in the phyllosphere via spraying, and in the stem by infiltrating. These modes of application can be effective in herbaceous plants and shrubs due to the small canopy and root area.

Phage viability is compromised in the phyllosphere. In a previous study by Balogh [107], phage concentrations at 10^4^ PFU/g of leaf tissue were found to be ineffective (Supplemental Table S1). In a field study, bacteriophage PFUs plummeted below 10^4^ PFU/g in the tomato phyllosphere within 4 h post-application applied prior to periods of high UVA + B in May and June in Florida, USA [80], indicating a loss of effective residual activity for disease control. Furthermore, phage residual activity was not detected the following day for those treatments. In contrast, an earlier study has shown that copper treatments exhibited residual efficacy on the tomato phyllosphere for at least 7 days after application [108]. Hence, the application of phage for disease control can be challenging compared to copper-based compounds in large areas, given that phage needs to be applied more frequently to maintain effective residual activity.

Additionally, spraying large trees or vines uniformly can be physically challenging [109]. One way to combat this is the application of phage through the vascular system [110]. For example, XylPhi-PDâ is a commercially available grapevine vascular injection that can be used to control Pierce’s disease of grapes caused by the vascular bacterial pathogen, *Xylella fastidiosa* subsp. *Fastidiosa* [111].

### 2.5. Possible Development of Phage Resistance in Bacterial Host

In nature, bacteria constantly face attacks from phages and have evolved a diverse array of defense mechanisms to counteract these infections. In response, phages have developed counterstrategies to evade bacterial defenses, leading to a dynamic evolutionary arms race between phages and their hosts [112]. This ongoing coevolution presents a major challenge for phage therapy, as bacterial resistance mechanisms can limit the long-term efficacy of phage-based treatments. Phage resistance in bacteria is a multifaceted process, driven by genetic adaptations, phase variation, and molecular modifications, which collectively enable bacteria to evade or neutralize phage infections [110,111].

One of the most common resistance mechanisms is blocking phage adsorption by altering the bacterial surface receptors. In many Gram-negative phytopathogens, this often involves mutations in cell envelope components such as lipopolysaccharides (LPS) or other outer-membrane structures that serve as phage receptors [113]. For example, the potato pathogen *Dickeya solani* can become resistant to lytic phage ΦD5 through mutations in genes required for LPS core or O-antigen synthesis. In one study, random Tn5 insertions in *D. solani* yielded multiple phage-resistant mutants, all showing small but effective modifications in their LPS structure that completely blocked ΦD5 adsorption. Notably, gel analysis revealed no major LPS band differences, suggesting that even minor structural modifications were sufficient to prevent phage binding. Similar LPS biosynthesis mutations conferring phage resistance have been reported in soft-rot *Pectobacterium* species (*P. atrosepticum*, *P. carotovorum*, and *P. brasiliense)* [113]. Likewise, in *Xanthomonas oryzae* (rice blight bacterium), spontaneous mutations in a glycosyltransferase gene involved in LPS assembly were shown to reduce phage adsorption by altering the O-antigen structure [114].

Another illustrative case is *Pseudomonas syringae*, a foliar plant pathogen. Laboratory experiments showed that phage-resistant *P. syringae* mutants carried mutations in the *rfbA* and *rfbD* genes, which are essential for LPS biosynthesis. Loss or alteration of these components eliminated the phage receptor, rendering the bacteria resistant to infection. However, this resistance comes at a cost: while mutants exhibited normal growth in rich media, they showed reduced virulence on tomato plants, likely due to the impaired LPS affecting bacterial fitness. Such fitness trade-offs are common in bacteria that evade phages through receptor loss. For example, transposon knockouts in *P. carotovorum* resulted in phage-resistant mutants that lost some ability to cause potato tuber rot [113].

Beyond surface modifications, phytopathogenic bacteria can acquire novel genetic elements that actively counteract phage infections. One of the most well-studied genetic resistance mechanisms is the CRISPR-Cas system, an adaptive immune mechanism found in many bacteria, including phytopathogens. The CRISPR-Cas system allows bacteria to capture short DNA fragments (spacers) from invading phages and store them in their genome. These spacers are later used as a molecular “memory” to recognize and degrade future phage infections [113,115,116,117]. The CRISPR-Cas system was first identified in yogurt bacteria (*Streptococcus thermophilus*) where it provided immunity against lytic phages, and subsequent studies have shown it can impose a fitness cost in some contexts (e.g., slowed growth due to the metabolic burden of maintaining the system) [118,119]. In phytopathogens, CRISPR-Cas loci are likewise present—for instance, *P. atrosepticum* carries multiple CRISPR spacer arrays—and they likely contribute to phage resistance by similar spacer acquisition and phage DNA targeting [117] (though detailed examples in planta are still emerging). In addition to CRISPR, bacteria often harbor or acquire other phage defense systems encoded by specific genes. Restriction-modification (R-M) systems involve restriction enzymes that cleave foreign (phage) DNA at specific sequences, paired with methylases that protect the host’s own DNA. R-M systems can be horizontally transferred on plasmids or mobile genetic elements, providing immediate resistance to many phages by degrading their genomes upon entry [120,121,122]. Another strategy is through abortive infection (Abi) systems, where a bacterium sacrifices itself upon phage invasion by triggering a self-destruct pathway, thereby aborting the phage replication cycle and protecting clonal bacterial neighbors. Genes for various Abi systems (such as toxin-antitoxin modules or phage trigger sensors) can be found on plasmids, transposons, or prophages in many bacteria [120]. In the context of plant pathogens, these horizontally acquired defense genes have not been studied as extensively as receptor mutations, but they likely operate under the surface. For example, some *Xanthomonas* and *Pseudomonas* strains possess plasmid-borne endonucleases and other defense proteins that could target phage DNA (paralleling what has been observed in human-pathogenic bacteria. By understanding the specific resistance mechanisms (and their costs) in phytopathogens, we can better design phage applications that either circumvent resistance or exploit the trade-offs associated with it [121].

### 2.6. Regulatory Issues

Regulatory authorities classify bacteriophages as biological substances, placing them under the purview of pharmaceutical legislation. Due to their classification as biological substances, they are subject to regulations mandating extensive data collection on safety, efficacy, and any potential environmental impact. In the European Union, the regulatory framework mandates that medicinal products produced through industrial processes require marketing authorization. This entails demonstrating safety, efficacy, and quality, with manufacturing conducted under Good Manufacturing Practices (GMP) [122]. However, achieving GMP compliance involves significant financial investment, posing a major barrier for new entrepreneurs in new phage product commercialization [123]. Legislation also demands rigorous qualitative and quantitative assessments of all components within a medicinal product. For phages, these criteria include the absence of prophages and antibiotic resistance in host bacteria, the exclusive lytic activity of phages against target pathogens, and stringent controls for impurities such as endotoxins and residual reagents. Given the limitations of the current regulatory framework, individual Member States within the European Union are adopting national-level strategies to address the regulation of phage therapy [124].

For phages targeting plant pathogenic bacteria, they must be approved for use as a biopesticide by the regulatory bodies overseeing a particular region [125]. In the United States, the Environmental Protection Agency evaluates potential new biopesticides under the guidance of the Federal Insecticide, Fungicide, and Rodenticide Act (FIFRA) to determine any potential risks to both human health and the health of the environment. The potential biopesticide or phage treatment must also meet the guidelines surrounding allowable levels of pesticide residues on the crop after harvest as outlined by the Federal Food, Drug, and Cosmetics Act (FFDCA) and the Food Quality Protection Act (FQPA). The Endangered Species Act also sets guidelines on biopesticide use to avoid off-target effects of pesticide use on any endangered species. Once a new phage treatment or biopesticide has gone through all the necessary evaluations, it can be approved for use on crops in the fields. As of now, only a few phage treatments have been approved as biopesticides for agricultural use by the EPA as of now.

## 3. Innovation over the Century to Tackle Challenges

### 3.1. Controlled Delivery Strategies

The application of bacteriophage-based biocontrol in agriculture faces significant challenges due to harsh environmental conditions, including UV radiation, desiccation, temperature fluctuations, and enzymatic degradation [81]. Unlike controlled environments in medical and veterinary settings, where pH fluctuations and enzymatic degradation within mammalian systems are the primary concerns, agricultural phage applications must contend with variable and often extreme abiotic stressors that can reduce phage viability and efficacy.

To overcome these challenges, advanced controlled delivery systems have been developed to enhance phage stability and effectiveness. These systems offer several advantages, including increased stability, localized and sustained availability, protection from UV degradation, improved adherence to target sites, and precision delivery to sites where the target organism resides. One of the most promising approaches for phage stabilization is encapsulation, where phages are embedded within protective matrices such as alginate, liposomes, chitosan, or biodegradable polymers. This method shields phages from environmental stressors, including UV exposure, desiccation, pH extremes, and enzymatic degradation, thereby improving storage stability, eliminating dependence on cold-chain logistics, and enabling phages to withstand manufacturing and field application stressors [126,127]. Recent studies have demonstrated the effectiveness of nano formulations in improving phage persistence in agricultural environments. For example, Nano N-Acetylcysteine-Zinc Sulfide has been used to enhance the phyllosphere persistence of phages, leading to a 16.4% reduction in bacterial spot disease severity in tomatoes [128].

The prospect of using bacteriophages for plant disease control is promising. The efficacy of bacteriophage can be improved by increasing stability in adverse environment (UV exposure, high pH, and temperature), improving the delivery and release methods, and increasing bactericidal activity by encapsulating the phages with different components. The following are substances used for coating bacteriophages to increase their effectiveness:

#### 3.1.1. Nanomaterials

A material is classified as a nanomaterial if its size or one of its dimensions is in the range of 1 to 100 nm [129]. Various nanomaterials have been tested for their ability to enhance the stability of bacteriophages. In a study by Choudhary et al. [128], nano–N–Acetylcysteine–Zinc Sulfide (nano-NAC-ZnS) formulated phage Φ*Xp*06-02-1 was assessed for its persistence in UV light in vitro and phyllosphere. In the study, phage persistence in the phyllosphere was 15-fold higher when formulated with nano-NAC-ZnS compared to non-formulated after 8 h of sunlight exposure. Additionally, nano-NAC-ZnS had some bactericidal effect after 24 h of incubation against two strains of *Xanthomonas euvesicatoria* pv. *perforans*, known to cause bacterial spot of tomato, help the phage for disease control.

Encapsulation of phage with nanomaterials has also shown promise in protecting them from extreme temperatures and pH conditions. Phage HK6, effective against *Enterobacter cloacae*, exhibited enhanced bactericidal activity and improved thermal and pH stability when encapsulated with chitosan nanoparticles (CS-NPs) [130]. Both encapsulated and free phages displayed similar stability at 25 °C, 50 °C, and 60 °C, as well as within a pH range of 5–12. However, encapsulation significantly improved phage stability at higher temperatures (70 °C and 80 °C) and extreme pH levels (3, 11, and 12).

Similarly, T7 phage engineered with a silver nanoparticle-binding peptide in capsid, when armed with silver nanoparticles, significantly reduced *E. coli* biofilm compared to phage alone [126]. This experiment also confirmed the non-toxicity of the recombinant phage bound with silver nanomaterials to eukaryotic cells, confirming its safety. Additionally, phage, PEL1, immobilized onto Fe_3_O_4_-based magnetic colloidal nanoparticle clusters coated with chitosan (PELI-CS-Fe_3_O_4_), demonstrated a significant reduction in mixed biofilms of *P. aeruginosa* and *E. coli* compared to PELI1 or CS-Fe_3_O_4_ alone [127], highlighting the synergistic effect of nano formulations with phage to manage bacteria.

#### 3.1.2. Hydrogels

**Figure 3:**
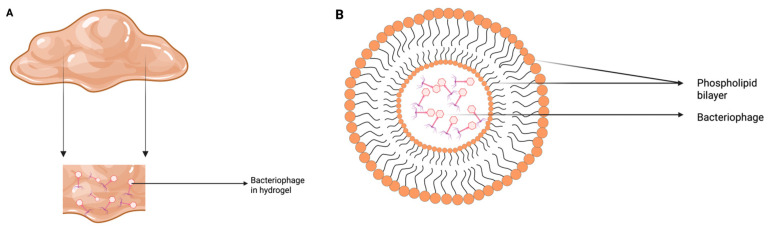
Bacteriophage encapsulation methods designed to protect phages from UV radiation and other environmental stresses in order to enhance phage stability and efficacy: (**A**) Hydrogel, (**B**) Liposome. These figures were inspired by Durr and Leipzig [142] and recreated by the authors.

Hydrogels are highly absorbent, 3D crosslinked polymer networks that retain large amounts of water while providing softness, flexibility, durability, and biocompatibility [131]. They can serve as a controlled delivery system for phages (Figure 3A) in targeted tissues by adjusting the porosity of the gel [132]. Co-delivery of phages has been successfully tested with various hydrogels, including carboxymethyl cellulose (CMC) [133,134] and alginate hydrogel [135]. Additionally, bacteriophage encapsulated with PEG-4MAL (poly (ethylene glycol)-4-maleimide) hydrogel was used to treat murine radial segmental defects infected with *P. aeruginosa* in mice without compromising the metabolic activity of human mesenchymal stromal cells or the bactericidal activity of the phage [136]. Notably, a 4.7-fold reduction in *P. aeruginosa* count was observed at the infection site by using phage treated with hydrogels compared to hydrogels alone, highlighting their potential for targeted antibacterial therapy.

#### 3.1.3. Liposomes for Encapsulation of Phages

Liposomes are microscopic, laboratory-made vesicular structures composed of single or multilayered phospholipid bilayers enclosing an aqueous compartment [137]. Their phospholipid layer can merge with cell membranes, facilitating the controlled release of their contents [138]. Liposomes have been used in human [139] and animal systems for drug delivery [138]. Encapsulation of the bacteriophage UAB_Ph87 in liposomes (Figure 3B) significantly enhanced its stability, increasing its resistance by 3.5-fold in simulated gastric fluid (pH 2.8) after 60 min of incubation compared to the non-encapsulated phage [140]. Furthermore, in a burn wound infection with *Klebsiella pneumonia* B5055 in mice, treatment with a phage cocktail loaded in liposomes resulted in a lower bacterial load in the skin, blood, and liver compared to liposomes alone or untreated control. This suggests that liposome encapsulation enhances the bactericidal activity of phages [141].

### 3.2. Genetic Engineering for Phages

Engineered bacteriophages offer a promising alternative to traditional antibiotics by overcoming key challenges such as host specificity, bacterial resistance, and immune system evasion. These phages can be tailored to degrade bacterial biofilms, evade bacterial defense mechanisms, and deliver antimicrobial payloads, making them a valuable tool in combating antibiotic-resistant infections [143,144]. A range of genome editing technologies has been employed to engineer bacteriophages for diverse applications. These technologies include homologous recombination [145], CRISPR (Clustered regularly interspaced short palindromic repeats) system [143], BRED (bacteriophage recombineering of electroporated DNA) [146], and in vivo recombineering [144]. These techniques have expanded phages’ potential applications in targeted bacterial eradication and microbiome modulation.

One notable example is phage Y2, a lytic phage targeting *Erwinia amylovora*, the fire blight pathogen. Since *E. amylovora* produces an exopolysaccharide (EPS) shield that hinders phage infection, Born et al. [147] enhanced Y2’s antimicrobial activity by introducing the *dpoL1* depolymerase gene via homologous recombination, creating the engineered phage (Y2::dpoL1-C) to degrade the EPS barrier. This modification enabled Y2 to degrade the EPS barrier, significantly enhanced bacterial killing, reducing *E. amylovora* counts in lab and field conditions, and preventing colonization of apple blossoms.

Another key advancement in phage engineering involves modifying host specificity through altering receptor-binding proteins. Mahichi et al. [148] successfully swapped the tail fiber gene of coliphage T2 with that from phage IP008, enabling T2 to infect a new host. Similarly, Marzari et al. [149] fused a receptor-binding domain from phage, broadening its host range.

To counteract bacterial resistance mechanisms, Qin et al. [150] engineered *Pseudomonas* phages to carry anti-CRISPR genes (AcrIF1, AcrIF2, AcrIF3), effectively disabling the bacterial CRISPR-Cas system and increasing phage infection success. Additionally, Yehl et al. [151] developed phage libraries with diverse tail fiber mutants, preventing the emergence of resistant bacterial clones and ensuring long-term efficacy in biocontrol strategies. These genetic modifications enhance phage efficacy, providing a powerful tool for sustainable biocontrol in agriculture. These approaches can be applied in agriculture to target specific phytopathogens or expand phage coverage across multiple bacterial species, improving biocontrol efficacy while minimizing bacterial resistance.

### 3.3. Combining Phages with Other Disease-Control Strategies for Managing Bacterial Diseases

Bacteriophages have been reported to be an effective biocontrol against several plant bacterial diseases (Table 1) and are a good alternative to antibiotics, as phages are abundant, highly specific, and have fewer environmental concerns. However, to improve the efficiency of phages in phage therapy and minimize phage resistance by bacterial pathogens, research studies have been geared toward supporting phage therapy with other chemical substances, thus combining phages with other disease control strategies (Table 2). Two studies [152,153] showed that a combination of bacteriophage and Systemic Acquired Resistance (SAR) inducers, such as acibenzolar-*S*-methyl (ASM), significantly reduced bacterial spot disease and gave more efficient disease control than phage alone or SAR alone. In another study, Abrahamian et al. [154] reported that bacteriophage applications against *X. perforans* were only effective in the field when in combination with other treatments such as copper octanoate (bactericides) and ASM (SAR inducers). Furthermore, treatments containing bacteriophage mixtures with plant activators or copper were compared for a more sustainable and effective control of *Xanthomonas axonopodis* pv. *allii* causing *Xanthomonas* leaf blight of onion [55]. They showed that the integration of bacteriophages with inducers such as acibenzolar-*S*-methyl (ASM) was equally effective or superior to the integration of phages with copper in reducing the disease severity. However, the use of bacteriophages with plant activators may produce a more viable option than using copper products, since plant defense stimulators are more sustainable, and they readily degrade in the ecosystem [155,156]. Not many studies have explored the combination of phages with other strategies; however, the combination of phages with other treatments should be aimed at reducing pathogen populations or improving the environment for phages, while ensuring the other treatments do not negatively affect the phage virions or interfere with phage replication [157]. In vitro assays, adding chemical or biological agents to phages to improve the lysis of target bacteria, further show potential IPM that can be used in field studies. Carvacrol combined with cocktail phages provided a higher efficiency of lysing *Pseudomonas syringae* pv. *actinidae,* the pathogen causing bacterial canker of kiwifruit, than using cocktail and individual phages alone [158]. *Pantoea agglomerans* served as a carrier strain for Erwinia phages and improved the concentration and in vitro lysis efficiency of *Erwinia amylovora* [159,160]. Furthermore, Kim et al. [161] showed a synergistic effect of cocktail phages with the antibiotic, kasugamycin, which resulted in a significant reduction in Erwinia *amylovora* in vitro and in immature wound-infected apples. These are potential IPM strategies that could be explored when using phages as biocontrol agents.

## 4. What Are the Practical Considerations Before and After Use?

The poor persistence of phages in the phyllosphere and rhizosphere leads to another important practical consideration when using phages for biocontrol. Previous studies have found a sharp decline in phage titer over only a few short hours, due to the deleterious conditions present in the phyllosphere, such as UV radiation, high temperatures, and exposure to chemical pesticides. Iriarte et al. [80] found that a phage targeting *Xanthomonas perforans* titers of 10^6^ or 10^8^ PFU/mL were able to reduce bacterial spot on tomatoes inoculated with 10^8^ CFU/mL of *Xanthomonas perforans*. However, phage titers of 10^4^ PFU/mL or lower were unable to reduce bacterial infection [107]. This decrease in titer also occurs naturally over a few hours, meaning that phage suspensions will quickly become ineffective in a short time after they are originally applied. Optimal timing of bacteriophage treatments, therefore, varies depending on the bacterial disease being treated and the environment of the bacteriophage application. It is difficult to describe overall best practices for the timing of phage applications; in general, however, it appears to be most effective to apply the phage treatment directly before bacterial inoculation.

Practically speaking, this means that phage suspension must be applied to the crop shortly before the bacterial species enters the plant. Given that it is impossible to determine exactly when a particular pathogenic bacterium will enter plants in the field, this means that the best chance of control comes from applying the phage suspension frequently. While a daily application may not be necessary for a particular pathosystem, phage concentrations tend to fall dramatically after 24 h, and so the phages should be applied frequently. Applying the phage suspensions in the evening hours aids longevity and persistence of phage activity on the plant, since that limits damage from UV radiation and higher temperatures during the day [15].

## 5. Conclusions

Bacteriophage therapy has undergone a remarkable evolution since its inception over a century ago, emerging as a viable alternative to traditional antimicrobial strategies, particularly in the face of rising antibiotic resistance. While its high specificity offers precision in targeting bacterial hosts, it simultaneously poses challenges related to narrow host ranges, resistance development, and practical implementation. Nevertheless, innovative advancements such as phage cocktails, genetic engineering, and novel delivery mechanisms have significantly expanded the scope and applicability of phage therapy across medicine, agriculture, and biotechnology.

To maximize the potential of phage therapy, a deeper understanding of phage–host dynamics is crucial, particularly in diverse and complex environments such as the rhizosphere, phyllosphere, and human microbiota. Research should also focus on the synergistic integration of phages with other biocontrol agents and therapeutic approaches to ensure both effectiveness and sustainability. Advances in genetic engineering can enable the customization of phages for broader host ranges, improved stability, and enhanced efficacy against resistant strains, potentially revolutionizing their use in both plant and human health.

In agricultural applications, overcoming challenges related to phage stability under harsh environmental conditions, large-scale application logistics, and regulatory compliance remains critical. Addressing these issues will require collaboration among researchers, industry stakeholders, and policymakers to establish standardized protocols, streamline approval processes, and develop cost-effective production and storage methods. Furthermore, the role of bacteriophages in shaping microbial ecosystems and their evolutionary interplay with bacterial hosts underscores their ecological importance beyond therapeutic applications. As research expands into uncovering novel phage types and mechanisms, the potential for groundbreaking discoveries in phage biology remains vast.

Phage therapy represents a promising paradigm shift toward precision-targeted microbial management. Its success, however, will depend on sustained investments in interdisciplinary research, innovation in delivery systems, and global regulatory frameworks that facilitate its safe and effective use. By addressing these multifaceted challenges, bacteriophage therapy can become a cornerstone in the fight against bacterial diseases, offering sustainable and adaptable solutions for global health and food security.

## Figures and Tables

**Table 1:** Summary of phages and their usage against plant bacterial diseases.

Pathogen	Phage Family ^a^	Disease Name with Host	Phage Conc. (PFU/mL)	Research Findings	Year of Study	References
*Acidovorax citrulli*	*Myoviridae*, *Siphoviridae*	Bacterial fruit blotch of melon	10^9^	Phage application in soil after symptom development resulted in 27% disease severity, compared to 80% for the non-treated control.	2020	[31]
*Dickeya solani*	*Myoviridae*	Potato soft rot	10^10^	Seed tuber treatment with phage led to a 13% yield increase and 5% reduction in disease incidence.	2012	[32]
*D*. *solani*	n/a	Blackleg of potato	10^6^–10^8^	Greater efficacy in reducing infection intensity was achieved in plants when higher concentration (10^8^ PFU/mL) of two-phage cocktails were applied on wounded tubers than treatments of lower concentrations. Preventative application of phage cocktail resulted in a decrease in infection intensity of 86.7% than 54.6% in curative application during field experiments.	2024	[33]
*Erwinia amylovora*	*Myoviridae*	Fire blight of pear and apple	10^8^–10^9^	Combination of phage with carrier bacterium (*Pantoea agglomerans*) reduced infection of detached pear tree blossoms by 84 to 96%.	2011	[34]
*E. amylovora*	*Myoviridae*	Fire blight of pear	10^8^	Stem injection of one-year old pear plants with a single phage developed no fire blight symptoms similar to plants treated with antibiotics, as compared with untreated plants that showed wilting, scorching and die-back symptoms, typical of fire blight.	2022	[35]
*E. amylovora*	*Myoviridae*	Fire blight of fruits	10^8^	Preventative inoculation of single phages 24 hrs before pathogen inoculation on detached fruits significantly reduced disease severity up to 66.7–100% than co-application of single phage with the pathogen. Two- to four-phage cocktail improved performance of phages in controlling incidence and severity.	2024	[36]
*E. amylovora*	n/a	Fire blight of fruits (blossom blight phase)	10^8^	Blossom blight incidence in field trials was significantly reduced in novel Erwinia phages and AgriPhage treatment compared with the unsprayed control with disease reductions ranging from 68.5 to 82.7%.	2024	[37]
*E. amylovora*	n/a	Fire blight of pear	10^7^	Preventative application of single phages on detached pears by wounding resulted in significantly smaller fire blight lesions as compared to untreated controls that showed clear fire blight symptoms. Application of cocktail phages drastically reduced necrosis than individual phage treatments.	2025	[38]
*Pectobacterium atrosepticum*	*Podoviridae, Myoviridae*	Soft rot of potato	10^8^	Use of the phage cocktail seed treatment reduced both disease incidence and disease severity by 61% and 64%, respectively.	2019	[21]
*P. carotovorum* subsp. *carotovorum,* *P. wasabiae and* *D.solani*	*Podoviridae, Myoviridae*	Soft rot of potato	10^5^	Application of individual or combined phages reduced soft rot severity caused by co-inoculation of the three soft rot pathogens, by 80% in potato slices and by 95% in whole tubers.	2015	[39]
*P. carotovorum* subsp. *carotovorum*	*Podoviridae, Siphoviridae*	Soft rot of onion	10^6^–10^8^	Immersion and spray inoculation of cocktail of four phages significantly reduced rot disease as compared to untreated controls.	2020	[40]
*Pectobacterium* spp.	*Straboviridae,* *Demerecviridae*	Soft rot of Chinese cabbage	10^8^	Pretreatment of individual phage or a two-phage cocktail significantly reduced soft rot symptoms by 60–95% in detached mature leaves, but the phage cocktail had greater control efficacy, which was as effective as commercial antibiotics. While pretreatment with individual phage or a phage cocktail on seedlings reduced symptom severity but was not as effective as the antibiotics.	2024	[41]
*Pseudomonas syringae* pv. *porri*	*Myoviridae*	Bacterial blight of leek	10^7^–10^8^	Application of cocktail phage decreased symptom development but could not completely stop infection.	2016	[42]
*P. syringae* pv. *actinidiae*	*Podoviridae*	Bacterial blight of kiwifruit	10^8^	Phage mixtures reduced bacteria load on kiwifruit leaves 24h post-infection by more than 75% in comparison with the untreated plants.	2020	[43]
*P. syringae* pv. *syringae*	*Podoviridae, Myoviridae*	Bacterial canker of cherry	10^8^	Phage cocktails reduced bacterial infection of alternate host (bean leaves) by almost 10-fold. Individual, 13- and 7-cocktail phages also significantly reduced bacterial populations on cherry leaves and twigs almost immediately after application compared to untreated controls.	2020	[44]
*P. tolaasii*	*Siphoviridae*	Brown blotch of mushroom	10^6^	Blotches on mushroom were completely blocked by co-incubation of phages with the pathogen.	2012	[45]
*Ralstonia solanacearum*	*Myoviridae*	Bacterial wilt of tomato	10^6^	Tomato plants pre-treated before pathogen inoculation with a single phage showed no symptoms of bacterial wilt.	2011	[46]
*R. solanacearum*	*Siphoviridae, Cystoviridae*	Bacterial wilt of potato	10^9^	Phage cocktail protected 80% of potato plants from wilt and reduced 98% of bacteria spiked in the sterilized soil at one week after spraying.	2017	[47]
*R. solanacearum*	*Podoviridae*	Bacterial wilt of tomato	10^11^	Phage treatment of tomato plants suppressed wilting by 100%, showing no disease symptoms compared to wilting in controls.	2018	[48]
*R. solanacearum*	*Podoviridae*	Bacterial wilt of tomato	10^6^	Phage cocktail application decreased the incidence of disease by up to 80% in field experiments.	2019	[20]
*R. solanacearum*	*Podoviridae*	Bacterial wilt of tomato	10^6^–10^10^	Co-inoculation of pathogen and single phage by irrigation significantly decreased bacterial wilt incidence in different biocontrol assays by up to 20–60%, compared to 80% in positive controls, while stronger reductions, or even absence of bacterial wilt incidence was achieved using two-phage or three phage cocktail mixtures.	2019	[49]
*R. solanacearum*	*Podoviridae*	Moko wilt of banana	10^7^	Two-phage cocktail provided 100% protection of plants from Moko disease, but plants treated with single phage treatment still displayed symptoms of the disease.	2020	[50]
*R. solanacearum*	*Myoviridae*	Bacterial wilt of tomato and potato	10^8^	Soil drenching of infected tomato seedlings with a single phage showed partial wilting symptoms, with a bacterial load reduction of 87%. Phage-treated tuber slices showed no lesion, with up to 81% reduction in bacterial population.	2021	[51]
*R. pseudosolanacearum*	*Autographiviridae*	Bacterial wilt of tomato	10^6^–10^8^	Treatment of tomato plants with single or cocktail phage at 10^8^ PFU/g soil significantly reduced bacterial wilt symptoms up to 40% as compared to control with up to 80% disease severity. Cocktail phages were only more effective at reducing disease severity than single phages at lower concentrations of 108 and 107 PFU/g soil.	2022	[52]
*R. solanacearum*	*Peduviridae*	Bacterial wilt of tomato	10^5^	Single application of four-phage cocktail significantly reduced disease incidence by 33–40%, while two- or three-times application of cocktail phage significantly reduced incidence to 67–84%, as compared to 100% in control treatment in greenhouse and field trials.	2024	[53]
*Streptomyces scabies*	*Siphoviridae*	Common scab of potato	10^9^	Seed tubers treated with phage resulted in tuber progeny with scab surface lesion of 1.2% compared with tubers harvested from non -treated seed tubers with 23%.	2001	[54]
*Xanthomonas* *axonopodis* pv. *allii*	n/a (AgriPhage)	Bacterial leaf blight of onion	10^5^–10^8^	Phage applications weekly and biweekly in field trials reduced disease severity similar to treatments of copper-mancozeb.	2007	[55]
*X. axonopodis* pv. *allii*	*Autographiviridae*	Bacterial leaf blight of onion	10^8^	Single lytic phage and a three-phage cocktail significantly reduced the progression of the disease; however, the single phage treatment provided higher disease protection and crop yield in field conditions.	2021	[56]
*X. campestris* pv. *pelargonii*	n/a	Bacterial blight of geranium	10^8^	Daily application of a mixture of four h-mutant phages on infected potted geranium and seedlings reduced disease incidence and severity significantly less than plants treated with phage mixture biweekly or triweekly.	2001	[57]
*X. campestris* pv. *campestris*	n/a	Black rot disease in crucifers	10^7^	Application of phage with non-pathogenic *Xanthomonas* strain on infected injured plants significantly improved the preventive effect against black rot symptoms. Field experiments using combination of phage with the non-pathogenic strain resulted in a 59% disease incidence than the positive controls.	2017	[58]
*X. campestris* pv. *campestris*	*Myoviridae*	black rot of crucifers	10^8^–10^9^	Irrigation-based application of individual phage on infected seedlings 14 days post inoculation of the pathogen was more effective to prevent disease development at 109 PFU/mL. Spray preventative application of phage was more effective in reducing disease than co-inoculation of phage and bacteria. Preventive application of the two-phage cocktail in field conditions led to a significant reduction in the number of symptomatic plants up to 67% compared to 96% in non-phage-treated plants.	2022	[59]
*X. campestris* pv. *campestris*	n/a	black rot of crucifers	10^7^	A single phage application on kohlrabi leaves showed reduced disease symptoms and late/no necrosis development as compared to the no-phage treatment controls having early and higher black rot symptoms with pronounced necrosis on leaves.	2025	[38]
*X. citri* subsp. *citri*	*Podoviridae*	Asiatic citrus canker	10^8^–10^10^	Greenhouse experiments utilizing phage treatment could reduce disease severity by 59% compared to controls.	2008	[16]
*X. citri* subsp. *citri*	*n/a*	Asiatic citrus canker	10^9^	Phage application with ASM showed 82.1 to 86.1% reduction in disease incidence.	2017	[60]
*X. euvesicatoria*	n/a (AgriPhage)	Bacterial leaf spot of tomato	10^10^	Greenhouse experiments with formulated phage cocktails could reduce disease severity and provide better protection to plants than unformulated phages.	2003	[15]
*X. euvesicatoria* pv. *citrumelonis*	*Podoviridae*	Citrus bacterial spot	10^8^	Phage treatments reduced citrus spot occurrence by 35 and 48% in two trials in commercial citrus nurseries.	2008	[16]
*X. euvesicatoria*	*Myoviridae*	Bacterial leaf spot of pepper	10^8^	Single phage treatments (pre- or post-pathogen inoculation) significantly reduced the lesion number on pepper leaves compared to the non-phage treated control. Double applications (pre- and post-pathogen inoculation) were not statistically different from single variant treatments.	2018	[61]
*X. oryzae* pv*. oryzae*	*Myoviridae*	Bacterial leaf blight of rice	10^8^	Phage formulation with skim milk significantly reduced the occurrence of BLB to 18.1% compared to 87% in untreated control.	2014	[62]
*X. oryzae* pv*. oryzae*	*Myoviridae*	Bacterial leaf blight of rice	10^8^–10^9^	Curative application of a single phage was most effective in reducing disease severity on rice plants at 2 days after pathogen inoculation than later days, but higher reduction was achieved in a preventative application by spraying (83.1%) and soaking of seeds with phage (95.4%).	2018	[63]
*X. oryzae* pv*. oryzae (Xoo), X. oryzae* pv*. oryzicola (Xoc)*	*Autographiviridae*	Bacterial leaf blight of rice (Xoo), bacterial leaf streak of rice (Xoc)	10^9^	The cocktail of two broad spectrum phages effectively reduced bacteria load in rice plants and significantly reduced the symptoms of respective disease caused by the pathogens. The Xoo phage cocktail had a better performance against Xoo and subsequently against disease symptoms than Xoc.	2023	[64]
*Xylella fastidiosa*	*Podoviridae,* *Siphoviridae*	Pierce’s disease of grapevine	10^10^	Pierce’s disease symptoms could be stopped using phage treatment post-infection as well as applying phage prophylactically to grapevines. Post and pretreatment with phage significantly reduced bacteria population (10 –1000 fold) compared to non-treated controls.	2015	[8]

^a^ The family names *Myoviridae*, *Siphoviridae*, and *Podoviridae* have been abolished, while *Autographiviridae and Peduoviridae* are some of the newly created family names according to the Current ICTV Taxonomy [65] n/a = not available in the literature.

**Table 2:** Use of phages with other chemical agents for integrated disease management.

Name of Diseases	Chemical Agents Used	Efficacy on Plant Diseases	References
Bacterial spot of tomato	acibenzolar-S-methyl (ASM)	Combination significantly reduced disease severity than the disease control achieved by only phage in field trials.	[152,153]
Bacterial spot of tomato	acibenzolar-S-methyl (ASM)and/copper octanoate	Combination was effective against disease severity in field trials as compared to the lone application of phage mixtures that reduced BST numerically but did not differ from non-treated control.	[154]
Bacterial spot of pepper	copper hydroxide	Combination with a single phage significantly reduced the lesion number on pepper leaves, same as copper-hydroxide treatments but was more effective than only bacteriophage treatments.	[61]
Bacterial spot of pepper	acibenzolar-S-methyl (ASM) and/copper hydroxide	Combination of both chemical agents with a single phage provided the most effective disease severity reduction and significantly higher yield than other integrated treatments with the phage.	[162]
Citrus canker	acibenzolar-S-methyl (ASM)	Combination reduced disease incidence significantly in greenhouse to 18.3% as compared to 75.2% in untreated control. Field trials also showed an 82.1% to 86.1% reduction in disease incidence.	[60]
Xanthomonas leaf blight of onion	acibenzolar-S-methyl (ASM), copper octanoate	Combination with ASM showed an improved efficacy, same or better than phage, in a single field trial location. However, with copper octanoate, the same efficiency was achieved as bacteriophages used alone.	[55]

## Supplementary Materials

The following supporting information can be downloaded at: https://www.mdpi.com/article/10.3390/v17081033/s1, Table S1: “Effect of phage concentration and copper-mancozeb treatment on bacterial spot disease development on tomato. Table from M.S. thesis by Balogh [102]”.
