# Supplementary material for: Bacteriophages as Agents for Plant Disease Control: Where Are We After a Century?
Source: Viruses. 2025 Jul 23;17(8):1033. doi: 10.3390/v17081033 (PMC12390404; doi:10.3390/v17081033)
Supplement: Supplementary file 1 [file viruses-17-01033-s001.zip › Bacteriophage supplemental 1.docx]

Supplemental Table 1: Effect of phage concentration and copper-mancozeb treatment

on bacterial spot disease development on tomato. Table from M.S. thesis by Balogh [73].

| **Treatment** | **Average lesion number^x^** |
| --- | --- |
| Skim milk (10^4^ PFU/ml phage) | 22.1^ab^ |
| Skim milk (10^6^ PFU/ml phage) | 13.8^c^ |
| Skim milk (10^8^ PFU/ml phage) | 16.2^bc^ |
| Non-formulated (10^8^ PFU/ml phage) | 17.1^abc^ |
| Copper-mancozeb | 5.1^d^ |
| Copper-mancozeb + Skim milk (10^8^ PFU/ml phage) | 2.6^e^ |
| Untreated control | 23.8^a^ |

^x^ Average lesion number per leaflet 16 days after inoculation. The mean’s followed by different letters within a column are significantly different according to Duncan’s multiple range test, P=0.05 level.

^y^ The constitution of treatments were skim milk – 0.75% powdered skim milk + 0.5% sucrose; non-formulated – deionized water only; copper-mancozeb – 3.2 g/l Kocide 2000 + 2.5 g/l Manzate 75DF.
